# Alterations of the Adipo–Myokine Irisin in Sepsis and Septic Shock: Diagnostic and Prognostic Implications

**DOI:** 10.3390/biom14030291

**Published:** 2024-02-29

**Authors:** Irene Karampela, Natalia G. Vallianou, Dimitrios Tsilingiris, Gerasimos Socrates Christodoulatos, Sotiria Psallida, Dimitris Kounatidis, Theodora Stratigou, Ioanna Marinou, Evaggelos Vogiatzakis, Maria Dalamaga

**Affiliations:** 1 Second Department of Critical Care, Attikon General University Hospital, Medical School, National and Kapodistrian University of Athens, 1 Rimini St., Haidari, 12462 Athens, Greece; 2 Department of Biological Chemistry, Medical School, National and Kapodistrian University of Athens, 75 Mikras Asias St., Goudi, 11527 Athens, Greece; madalamaga@med.uoa.gr; 3 Departments of Internal Medicine and Endocrinology, Evangelismos General Hospital, 45-47 Ipsilantou St., 10676 Athens, Greece; natalia.vallianou@hotmail.com (N.G.V.); dimitriskounatidis82@outlook.com (D.K.); theodorastratigou@yahoo.gr (T.S.); 4 First Department of Internal Medicine, University Hospital of Alexandroupolis, Democritus University of Thrace, 68100 Alexandroupolis, Greece; tsilingirisd@gmail.com; 5 Department of Microbiology, Sismanogleio General Hospital, 1 Sismanogleiou St., 15126 Athens, Greece; gerchristod82@hotmail.com; 6 Department of Microbiology, ‘KAT’ General Hospital of Attica, 2 Nikis St., 14561 Athens, Greece; psallidasotiria@gmail.com; 7 Laboratory of Microbiology, Sotiria Athens General Hospital, 152 Mesogion Ave., 11527 Athens, Greece; ioannachond@yahoo.gr (I.M.); vogia2@gmail.com (E.V.)

**Keywords:** adipokine, biomarker, critically ill, irisin, mortality, myokine, sepsis, septic shock

## Abstract

Irisin, a novel adipo-myokine with metabolic regulatory functions, exerts anti-inflammatory, antioxidant, and anti-apoptotic actions that may confer protection against sepsis-induced organ injury in experimental studies. Until now, only one human study has explored circulating irisin at sepsis onset. We aimed to examine serum irisin and its kinetics in critically ill patients with sepsis and septic shock with regard to sepsis severity and outcome. We enrolled 102 critically ill patients with sepsis or septic shock within 48 h of diagnosis and 102 age- and gender-matched healthy controls. Irisin was determined in serum upon enrollment in all participants and one week later in patients using an immunoenzymatic method. The outcome of sepsis was recorded 28 days after enrollment. At enrollment, circulating irisin was significantly lower in patients than controls (22.3 ± 6.8 μg/L vs. 28.1 ± 6.7 μg/L, *p* < 0.001), and increased significantly one week later (22.3 ± 6.8 μg/L vs. 26.6 ± 9.5 μg/L, *p* < 0.001). Irisin was significantly lower in patients who presented with septic shock than those with sepsis, and in non-survivors than survivors both at enrollment and one week later. However, kinetics of irisin did not differ between the groups (*p* > 0.05). Patients with higher circulating irisin during the first week of sepsis had a better outcome (*p* < 0.001). Lower irisin was independently associated with 28-day mortality (sepsis onset: HR 0.44, 95% C.I. 0.26–0.77, *p* = 0.004 and one week after: HR 0.37, 95% C.I. 0.23–0.58, *p* < 0.001). Irisin was negatively correlated with severity scores, metabolic, and inflammatory biomarkers. Circulating irisin decreases early in sepsis and is an independent predictor of 28-day mortality. Irisin may be a promising diagnostic and prognostic sepsis biomarker; nevertheless, larger studies are needed to explore its role in sepsis.

## 1. Introduction

Irisin is a recently described protein with cytokine-like actions mainly excreted by the muscle cells [1]. Its precursor is a membrane protein named Fibronectin type III domain-containing protein 5 (FNDC5), which is expressed and produced by muscles in response to exercise. Proteolytic cleavage of the extracellular fragment of the FNDC5 releases a 112 amino-acid sequence, which comprises irisin [1,2]. Following secretion, irisin enters the circulation, and it is found in plasma [3]. Irisin is also expressed and secreted in the adipose tissue; thus, it is considered an adipo–myokine [4,5]. It is also found in the liver, pancreas, stomach, spleen, and nerves [6].

Irisin is known as an exercise-induced hormone with autocrine, paracrine, and endocrine activity, exhibiting metabolic regulatory effects not only on the muscles but also on remote organs. Irisin exerts important physiological actions, as it: (1) induces browning of the white adipose tissue and increases thermogenesis and energy expenditure; (2) enhances glucose uptake in muscle cells and reduces insulin resistance; and (3) stimulates muscle growth and promotes neural and osteoblast differentiation [4,7,8,9,10,11,12]. Clinical studies have shown a protective effect of irisin against metabolic disorders, such as metabolic syndrome and obesity, type 2 diabetes mellitus (T2DM), dyslipidemia, cardiovascular diseases, nonalcoholic fatty liver disease, and metabolic bone diseases [13,14,15,16,17]. Moreover, irisin is implicated in tumor development and progression, being considered protective against various types of cancer [18,19].

Interestingly, in addition to metabolic regulation, irisin exerts anti-inflammatory actions through multiple signaling pathways [10,20,21,22]. Experimental studies have shown that irisin may induce the production of anti-inflammatory cytokines, while it may inhibit the secretion of pro-inflammatory cytokines and suppress inflammatory chemoattractant molecules [10,23,24]. Thus, irisin may attenuate the acute inflammatory response [25,26].

Sepsis, a life-threatening organ dysfunction caused by a dysregulated host response to infection, is characterized by an aberrant inflammatory response [27]. Sepsis bears a high mortality rate, greater than 10%. Moreover, its most severe form, septic shock, is associated with hospital mortality rates greater than 40%. Septic shock is a subset of sepsis, with particularly profound circulatory, cellular, and metabolic abnormalities and is defined as sepsis with persisting hypotension requiring vasopressors and having a serum lactate level >2 mmol/L (18 mg/dL) despite adequate volume resuscitation [27]. Early recognition and treatment may improve the outcome of sepsis and septic shock. However, reliable sepsis biomarkers are lacking. Currently used biomarkers of infection, such as C-reactive protein and procalcitonin, have modest diagnostic and prognostic value. Therefore, the study of novel biomarkers and their kinetics for early diagnosis and prognosis of sepsis is an important research area.

Recent experimental studies have demonstrated a protective role of irisin in sepsis-induced organ dysfunction through the inhibition of multiple inflammatory pathways [21,28,29,30,31,32,33]. However, irisin has not been thoroughly studied in humans, i.e., in patients with sepsis and septic shock. There is only one recent clinical study on patients with sepsis and another study on the expression of the irisin precursor FNDC5 in critically ill patients [34,35]. Moreover, no human study has examined irisin kinetics in the first week of sepsis. Therefore, we aimed to investigate circulating irisin in critically ill patients with sepsis and septic shock at the onset of sepsis compared to healthy controls. Furthermore, our goal was also to study the kinetics of serum irisin during the first week of sepsis and its associations with the severity and outcome of sepsis in a prospective study.

## 2. Materials and Methods

### 2.1. Study Design and Participants

In this prospective observational study, consecutive critically ill patients hospitalized in the Intensive Care Unit (ICU) of a tertiary teaching hospital during a two-year period were enrolled if they met the following inclusion criteria: (1) age ≥ 18 years; (2) diagnosis of sepsis no more than 48 h prior. We excluded patients according to the following criteria: (1) endocrine disease including T2DM; (2) liver disease; (3) total parenteral nutrition; (4) malignancy; (5) immunosuppression; and (6) pregnancy. Additionally, we retrospectively excluded patients who did not complete one week of hospitalization in the ICU after enrollment in the study. The diagnosis of sepsis and septic shock upon enrollment was made according to the 3rd International Consensus Definitions (SEPSIS-3) [27]. We recorded basic demographic and clinical data as well as main laboratory data during the first week of enrollment. All patients received the standard of care according to international guidelines. We also recorded the outcome of sepsis at 28 days from inclusion in the study. For every eligible patient, we enrolled a gender- and age-matched (±5 years) healthy adult as a control, recruited among visitors of the outpatient Laboratory Department of the hospital. The same exclusion criteria were applied for controls. Demographics and main laboratory data were recorded.

The study was conducted according to the guidelines of the Declaration of Helsinki and its successive amendments and was approved by the Scientific and Ethics Committee of the hospital (#587/10-04-2013). Informed consent was obtained from all subjects involved in the study or their next of kin. The study protocol has been previously published in detail [36].

### 2.2. Laboratory Evaluation

Whole blood samples were collected from patients and controls upon enrollment and also one week after inclusion in the study, but only in patients. All samples were centrifuged to extract the serum, which was then stored at −80 °C for future analysis. Circulating irisin was determined by an immunoenzymatic method using an ELISA kit (EK-067-29, Phoenix Pharmaceuticals, Burlingame, CA, USA), which is one of the best currently available and validated kits toward tandem mass spectrometry for irisin measurements, with a sensitivity of 1.7 ng/mL [37]. We also determined hematologic, coagulation, metabolic, and inflammatory parameters. Determination of interleukins (IL) IL-1β, IL-6, IL-10, and soluble urokinase-type Plasminogen Activator Receptor (suPAR) was conducted by ELISA (eBiosciences, San Diego, CA, USA and suPARnostic™, ViroGates, Lyngby, Denmark), as previously described [38,39]. A homeostasis model assessment score of insulin resistance (HOMA-IR) was calculated as follows: [fasting serum insulin (μU/mL) × fasting serum glucose (mmol/L)]/22.5.

### 2.3. Statistical Analysis

Assessment of categorical variables was performed by the chi-square test. The normality hypothesis was examined by the Shapiro–Wilk test. Analysis of normally distributed variables was performed by the *t*-test and paired *t*-test, while for not normally distributed variables, the Mann–Whitney U and Wilcoxon matched-pair tests were used. Multivariable binary logistic regression analysis was used to explore whether irisin was independently associated with disease status (sepsis patients versus controls, dependent variable), adjusting for age, gender, and BMI. Continuous variables were analyzed using the Spearman correlation coefficients (r) as a measure of correlation. The Kaplan–Meier method was used for survival analysis, while the log rank test was used for comparisons. In order to calculate the discriminating power of biomarkers for sepsis and septic shock, we evaluated Receiver Operating Characteristic (ROC) curves. The DeLong test was used to compare ROC curves. Multivariate Cox-regression analysis, adjusting for Acute Physiology and Chronic Health Evaluation II score (APACHE II) and statistically significant biomarkers, was performed for the determination of independent predictors of 28-day mortality. Based on previous studies on adipokines, we calculated that we required a total sample size of at least 200 participants to achieve 95% power at the 0.05 level of significance, in order to detect a 3 μg/L difference in circulating irisin [40,41,42,43,44,45]. A two-sided *p*-value of less than 0.05 was considered significant. The statistical package IBM-SPSS^®^ version 24 for Windows (Armonk, NY, USA: IBM Corp.) was used for the statistical analysis, and the MedCalc^®^ Statistical Software version 20.218 (MedCalc Software Ltd., Ostend, Belgium) was used for the DeLong test.

## 3. Results

### 3.1. Characteristics of Patients and Controls

From the initial 167 patients meeting the inclusion criteria, 65 patients were excluded as follows: 17 due to endocrinopathy; 7 due to malignancy; 5 due to immunosuppression; and 36 due to death or discharge in less than a week from enrollment. One hundred and two patients (57 males, aged 64.7 ± 15.6 years) and 102 healthy subjects (57 males, aged 66.4 ± 10.3 years) were included in the study. Sixty-one cases were medical, and 41 cases were surgical sepsis cases, including trauma patients. The most common sites of infection causing sepsis were the lungs at 35% and the abdomen at 24%. The most frequently identified infectious agents were Gram-negative bacteria (60%) and Gram-positive bacteria (23%), while fungi were identified in 17% of cases. Sixty patients presented with sepsis at enrollment and 42 with septic shock. Thirty patients died within 8 to 28 days after inclusion in the study.

Table 1 presents the demographic, clinical, and laboratory baseline data of patients and controls. Notably, BMI did not differ significantly between the two groups (*p* = 0.06). The hematologic, coagulation, organ dysfunction, main metabolic parameters, and C-reactive protein (CRP) were significantly different between patients and controls (*p* < 0.05), with the exception of creatinine (*p* = 0.08) [Table 1].

### 3.2. Circulating Irisin in Patients and Controls

**Figure 1:**
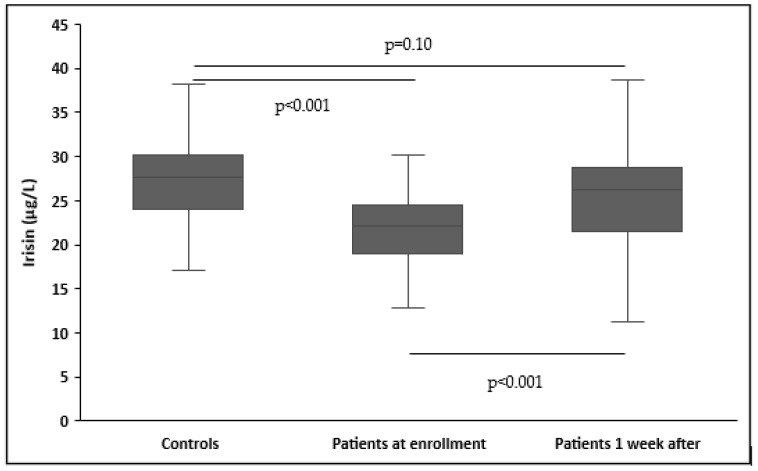
Circulating irisin levels in controls (N = 102) and patients (N = 102) at enrollment and one week after.

Upon enrollment, patients had significantly lower serum irisin levels than controls (22.3 ± 6.8 μg/L vs. 28.1 ± 6.7 μg/L, *p* < 0.001) (Table 1). The significant differences in irisin concentrations remained unaltered after adjusting for age, gender, and BMI (*p* < 0.001). In patients, no difference was found in irisin concentrations based on sepsis etiology (medical or surgical) (*p* = 0.32), site of infection (*p* = 0.6), causative pathogen (*p* = 0.29), and documentation of bacterial infection (*p* = 0.37). Furthermore, circulating irisin increased significantly in all patients one week after inclusion in the study (22.3 ± 6.8 μg/L vs. 26.6 ± 9.5 μg/L, *p* < 0.001). However, patients one week after enrollment did not have significantly decreased serum irisin levels than controls (26.6 ± 9.5 μg/L vs. 28.1 ± 6.7 μg/L, *p* = 0.10) (Figure 1).

### 3.3. Circulating Irisin in Sepsis and Septic Shock

**Figure 2:**
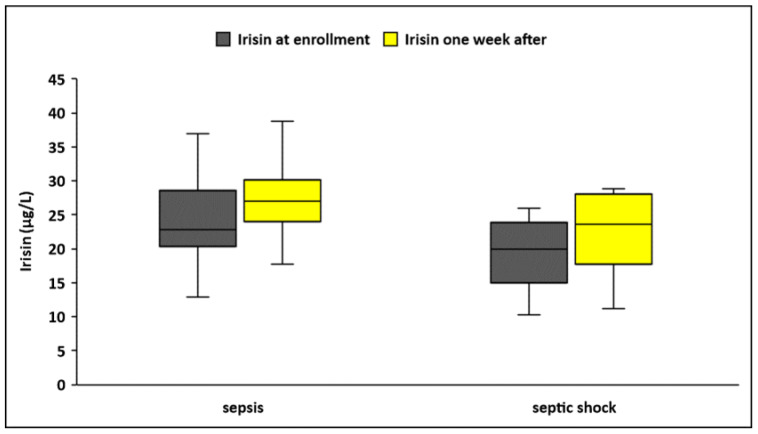
Circulating irisin in patients with sepsis (N = 60) and septic shock (N = 42) at enrollment and one week after.

Circulating irisin levels were significantly lower in patients who presented with septic shock at enrollment (N = 42) compared to those presented with sepsis (N = 60), both upon enrollment (19.6 ± 5.1 μg/L vs. 24.2 ± 7.3 μg/L, *p* < 0.001) and one week after (23.6 ± 7 μg/L vs. 28.7 ± 10.5 μg/L, *p* = 0.004) (Table 2, Figure 2). Irisin increased significantly during the first week of sepsis in both groups (septic shock, *p* = 0.002; sepsis, *p* < 0.001). However, kinetics of irisin did not differ between the groups. (Δirisin%: 25 ± 47 vs. 24 ± 56, *p* = 0.87).

**Figure 3:**
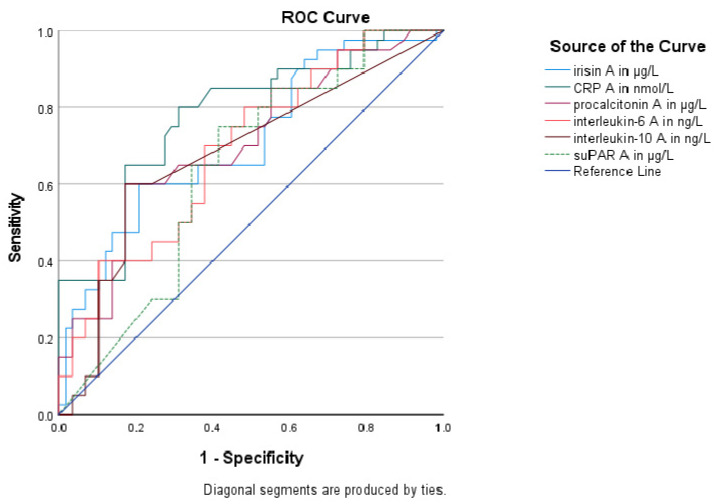
Receiver Operating Characteristic Curves of circulating irisin and inflammatory biomarkers to distinguish sepsis from septic shock in 102 patients.

**Figure 4:**
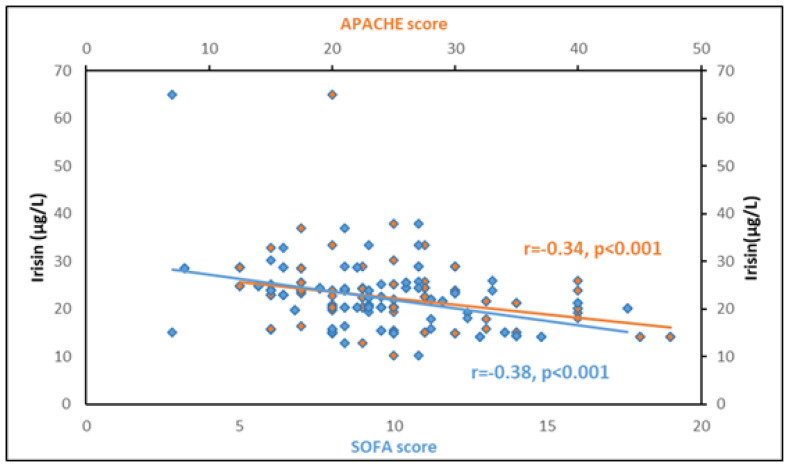
Circulating irisin is significantly associated with the severity scores APACHE II and SOFA at enrollment in 102 patients with sepsis and septic shock. The correlation between irisin and SOFA score is depicted in blue, while the correlation between irisin and APACHE score is depicted in orange.

The discriminating power of irisin and other inflammatory biomarkers for sepsis and septic shock was evaluated using ROC curves (Table 3). Circulating irisin (AUROC > 0.72), CRP (AUROC > 0.78), and procalcitonin (AUROC > 0.71) at enrollment presented superior discriminative ability (as expressed by areas under the ROC curves/AUROC) compared to IL-6 (AUROC > 0.69), IL-10 (AUROC > 0.68), and suPAR (AUROC > 0.64) in distinguishing sepsis from septic shock; nevertheless, the comparison of ROC curves did not yield any statistically significant results (*p* > 0.05 derived from the DeLong test) (Figure 3). Furthermore, circulating irisin at enrollment presented a significant negative association with the severity scores APACHE II and SOFA (r= −0.34, *p* < 0.001 and r= −0.38, *p* < 0.001, respectively) (Figure 4).

### 3.4. Circulating Irisin According to Sepsis Outcomes

**Figure 5:**
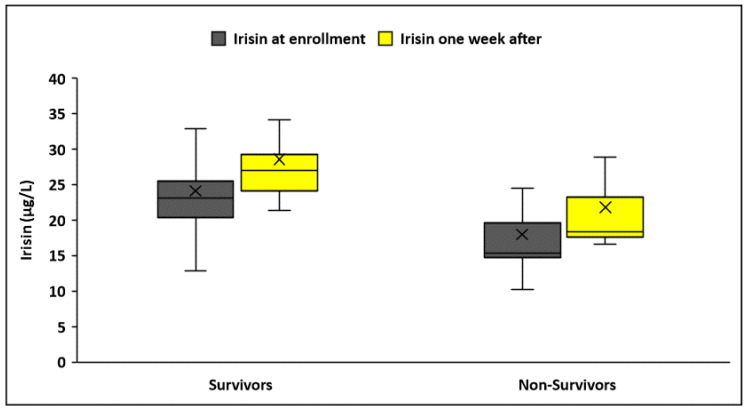
Circulating irisin in survivors (N = 72) and non-survivors (N = 30) during 28 days of follow-up, at enrollment and one week after.

**Figure 6:**
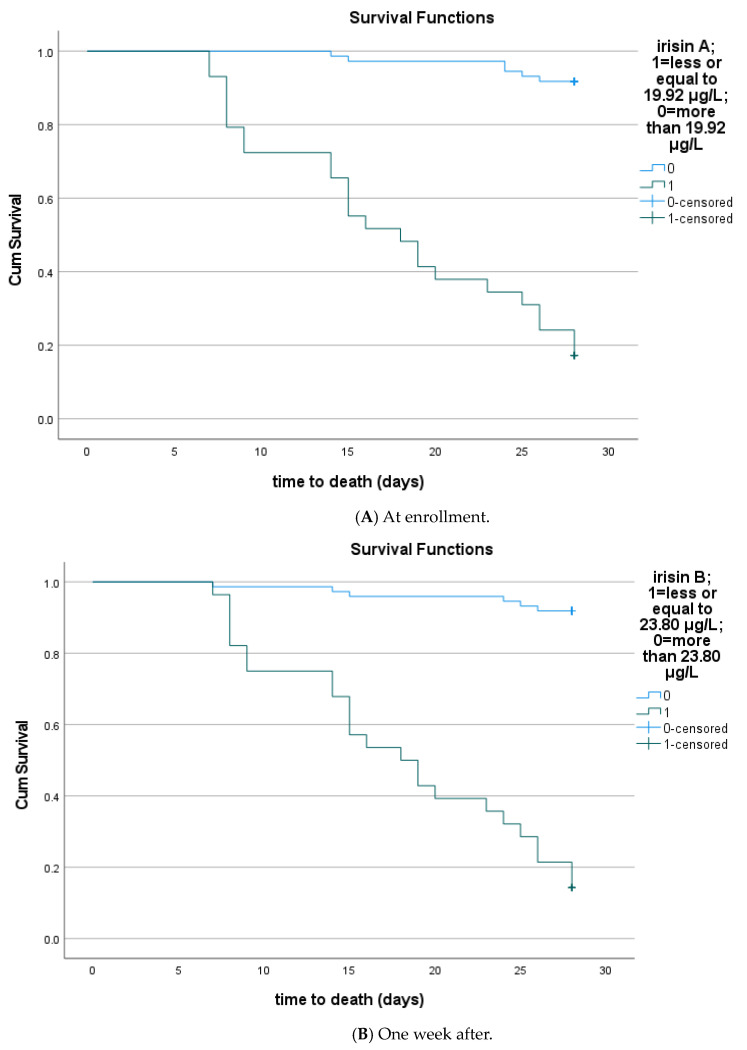
(**A**). Kaplan–Meier estimates of mortality in 102 septic patients based on circulating irisin at enrollment. Patients with elevated serum irisin levels (>19.92 μg/L) at enrollment had improved 28-day survival (log rank test: 76.89, *p* < 0.001). (**B**). Kaplan–Meier estimates of mortality in 102 septic patients based on circulating irisin one week after enrollment. Patients with elevated serum irisin levels (>23.8 μg/L) one week after had improved 28-day survival (*p* < 0.001) (log rank test: 79.25, *p* < 0.001).

Circulating irisin was significantly lower in patients who did not survive sepsis during the 28 days of follow-up after enrollment (N = 30) compared to survivors (N = 72), both at enrollment (17.9 ± 6.3 μg/L vs. 24.1 ± 6.2 μg/L, *p* < 0.001) and one week after (21.8 ± 7.8 μg/L vs. 28.6 ± 9.5 μg/L, *p* < 0.001) (Figure 5). Irisin increased significantly one week after sepsis onset only in survivors (*p* < 0.001). However, kinetics did not differ between the two groups (Δirisin%: 29 ± 56 vs. 22 ± 51, *p* = 0.27). Using logistic regression analysis, the recovery of irisin (irisin percentage change after one week) was not a significant parameter for the survival of patients (OR: 1.002, 95% C.I. 0.99–1.01; *p* = 0.53). The Kaplan–Meier survival curves showed that patients with higher irisin at enrollment and one week after had improved 28-day survival (*p* < 0.001) (Figure 6). The cutoff value of irisin was estimated at 19.92 μg/L at enrollment and 23.8 μg/L one week after enrollment. The cutoff values were obtained via ROC analysis of circulating irisin to distinguish death from survival, as depicted in Table 4. Circulating irisin (AUROC > 0.81) and CRP (AUROC > 0.72) at enrollment presented higher discriminative ability (as expressed by areas under the ROC curves/AUROC) than procalcitonin, IL-6, and IL-10 (AUROC > 0.65) in discriminating death from survival; nevertheless, the comparison of ROC curves did not yield any statistically significant results (*p* > 0.05, derived from the DeLong test).

Unadjusted Cox regression analyses demonstrated that circulating irisin at enrollment (HR: 0.41, 95% C.I. 0.24–0.69, *p* < 0.001) and one week after (HR: 0.32, 95% C.I. 0.20–0.51, *p* < 0.001) were significantly associated with 28-day mortality of sepsis. After adjustment for the APACHE II score and significant laboratory biomarkers, lower circulating irisin both at enrollment and one week after sepsis onset were independently associated with 28-day mortality (HR 0.44, 95% C.I. 0.26–0.77, *p* = 0.004 and HR 0.37, 95% C.I. 0.23–0.58, *p* < 0.001, respectively) (Table 5). Notably, neither CRP nor IL-6 at enrollment were independent predictors of 28-day mortality. However, higher IL-6 one week after sepsis onset was also independently associated with mortality (HR 1.68, 95% C.I. 1.13–2.49, *p* = 0.01).

### 3.5. Association of Circulating Irisin with Other Biomarkers

Circulating irisin at sepsis onset exhibited significant negative correlations with white blood cells, activated partial thromboplastin time (aPTT), and metabolic biomarkers (lactate, alanine and aspartate aminotransferases, and HOMA-IR) (Table 6). Additionally, baseline irisin was negatively correlated with major inflammatory biomarkers (CRP, procalcitonin, IL-6, and IL-10) but not IL-1β and suPAR. Of note, only irisin’s negative correlation with lactate and procalcitonin persisted one week after sepsis onset. Interestingly, irisin did not correlate with BMI in septic patients.

## 4. Discussion

In this prospective observational clinical study on 102 critically ill patients with sepsis or septic shock, we found that circulating irisin was significantly lower at sepsis onset compared to 102 gender- and age-matched healthy controls, independent from BMI. Irisin levels were not related to sepsis etiology (medical or surgical), the site of infection, or the causative pathogen. Regarding kinetics, irisin significantly increased one week after sepsis onset; however, its recovery (irisin percentage change after one week) was not a significant parameter of patients’ survival. We also found that circulating irisin was negatively associated with the severity of sepsis, being significantly lower in patients with septic shock compared to patients with sepsis both at sepsis onset and one week after. Irisin at sepsis onset exhibited a good discriminating ability for sepsis and septic shock, similar to the well-established biomarkers CRP and procalcitonin. Furthermore, we found that irisin at sepsis onset and one week after was significantly lower in non-survivors compared to survivors at 28 days after enrollment. Lower irisin during the first week of sepsis was significantly associated with 28-day mortality, being an independent predictor of mortality after adjustment for the APACHE II score.

There is only one small previous clinical study on circulating irisin in patients with sepsis [34]. In line with our findings, this study showed that serum irisin within 24 h of hospital admission was significantly lower in 60 patients with sepsis compared to 29 healthy subjects. It also showed that irisin was negatively associated with blood lactate and the APACHE II score, in agreement with our findings. Moreover, in another clinical study, serum irisin was significantly lower in 60 patients with acute respiratory distress syndrome (ARDS) compared to 60 healthy volunteers, and it presented significant negative correlations with APACHE II and SOFA scores, similar to our findings in patients with sepsis [26]. This study also showed that serum irisin levels were independently associated with 28-day mortality (HR, 0.153; 95% CI, 0.024–0.961; *p* = 0.045), in agreement with our study on sepsis. Although the authors did not report the incidence of sepsis in their patient cohort, it has to be emphasized that sepsis is the most frequent cause of ARDS. Finally, in a recent clinical study, mRNA expression of the irisin precursor FNDC5 in muscle biopsies was lower in 162 critically ill patients after 8 ± 1 days in the ICU compared to 19 healthy controls and was independently associated with ICU mortality [35]. Interestingly, 58% of these patients had sepsis upon admission.

Experimental studies have shown that, in addition to its metabolic effects, irisin exerts anti-inflammatory, antioxidant, and anti-apoptotic activities, suggesting that it may be a potent immunometabolic regulator with a protective role not only in chronic subclinical inflammatory conditions such as obesity and related metabolic disorders but also in acute inflammatory states such as sepsis [13,16,46]. In particular, irisin may suppress the secretion of inflammatory cytokines in serum, the lung, kidney, and heart, to reduce oxidative stress and restore mitochondrial function in septic mice [34].

**Figure 7:**
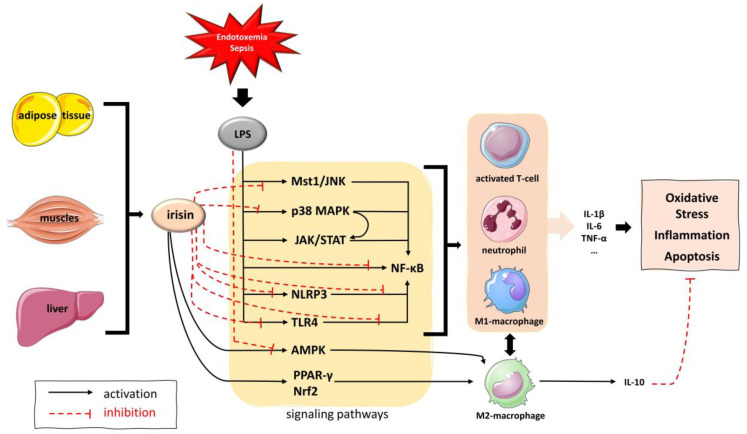
Potential pathophysiologic mechanisms that interfere with important inflammatory signaling pathways to mediate irisin’s anti-inflammatory, antioxidant, and anti-apoptotic actions. **Abbreviations:** AMPK, adenosine monophosphate-activated protein kinase; JNK, c-Jun n-terminal kinase; IL, interleukin; JAK/STAT, Janus kinase/signal transducers and activators of transcription; LPS, lipopolysaccharide; MAPK, mitogen-activated protein kinases; Mst1, macrophage stimulating 1; NF-kB, nuclear factor kappa B; NLRP3, NOD-like receptor protein 3; Nrf2: nuclear factor erythroid 2–related factor 2; PPAR-γ, peroxisome proliferator-activated receptor gamma; TLR4, Toll-like receptor 4; TNF-a, tumor necrosis factor alpha. (Free elements of the images are originated from the free medical site http://smart.servier.com/ (accessed on 20 January 2024) by Servier, licensed under a Creative Commons Attribution 3.0 Unported License).

Evidence from in vitro and in vivo experimental studies suggests that irisin acts through intricate signaling pathways, including the p38 mitogen-activated protein kinases (MAPK), the adenosine monophosphate-activated protein kinase-α (AMPK-α), the transcription factor nuclear factor kappa B (NF-kB), the Janus kinase 2 (JAK2), and the macrophage-stimulating 1 and Jun N-terminal kinase (Mst1-JNK) pathway, among others [20,21]. Additionally, irisin inhibits inflammatory cell migration and infiltration and reduces vascular permeability, leading to an attenuation of the acute inflammatory response [25,26]. Furthermore, irisin has been shown to favor the anti-inflammatory M2-type macrophage polarization over the inflammatory M1-type through activation of the peroxisome proliferator-activated receptor gamma (PPAR-γ)-related anti-inflammatory system and the nuclear factor-erythroid 2-related factor 2 (Nrf2)-related antioxidant genes [22]. Finally, irisin prevents the formation of inflammasome, thus, ameliorating the inflammatory response and promoting cellular viability [29,33,47] (Figure 7).

Regarding sepsis, only recently has evidence from in vitro and in vivo experimental studies on animal and cell models of sepsis shown promising results supporting a protective role of irisin in sepsis-induced organ dysfunction, such as septic cardiomyopathy, encephalopathy, and acute kidney, lung, and liver injury. Most of these studies have investigated sepsis-associated cardiac dysfunction. Tan et al. demonstrated that irisin treatment attenuated LPS-mediated cardiomyocyte death and myocardial dysfunction by inhibiting dynamin-related protein 1 (DRP1)-related mitochondrial fission through the Jun N-terminal—Large Tumor Suppressor 2 kinase (JNK-LATS2) signaling pathway in LPS-induced sepsis in mice [28]. Ouyang et al. reported that in a mouse model of sepsis, treatment with irisin and melatonin improved mitochondrial function and promoted cardiomyocyte viability through the inhibition of Mst1-JNK [21]. Moreover, in a case-control animal study of LPS-induced sepsis, Li et al. demonstrated that LPS promoted cardiomyocyte death (apoptosis and pyroptosis) and enhanced the expression of pro-inflammatory mediators through the Toll-like receptor 4 (TLR4) and the NF-kB signaling pathway and the formation of the inflammasome NOD-like receptor protein 3 (NLRP3). Irisin treatment suppressed inflammation, apoptosis, and pyroptosis by blocking the Toll-like receptor 4 (TLR4) and NLRP3 inflammasome signaling in vivo and in vitro and attenuated myocardial dysfunction in sepsis [29]. In line with the previous findings, Xiong et al. showed that irisin may protect against LPS-induced cardiomyocyte injury through the activation of mitochondrial ubiquitin ligase and the resulting inhibition of gasdermin D-dependent pyroptosis [48]. Finally, an in vitro study on LPS-stimulated cardiomyocytes showed that irisin significantly reduced oxidative stress by increasing the activities of antioxidant enzymes and inhibited cardiomyocyte apoptosis by suppressing the activation of caspase-3 and caspase-9 [49].

In vitro and in vivo studies also support the beneficial effects of irisin in sepsis-associated acute kidney, liver, and lung injury. A recent study showed that irisin treatment reduced NF-kB expression and reversed LPS-induced expression of pro-inflammatory cytokines, while it also decreased the apoptotic rate in renal tubular cells [32]. Moreover, irisin has been shown to suppress ferroptosis (a type of cell death elicited by iron-dependent lipid peroxidation) and protect from sepsis-associated acute kidney injury through activation of the sirtuin 1/Nrf2 (SIRT1/Nrf2) signaling pathway [31]. Furthermore, irisin was found to suppress ferroptosis and protect from sepsis-associated encephalopathy and liver injury in vivo and in vitro through the Nrf2/Glutathione peroxidase 4 (Nrf2/GPX4) signaling pathway [30,34]. Additionally, in an LPS-induced animal and cell model of liver injury, irisin inhibited apoptosis, NLRP3 inflammasome activation, and NF-kB signaling, and attenuated the release of inflammatory cytokines and liver injury [47]. Finally, irisin suppressed inflammation and apoptosis and improved LPS-induced alveolar epithelial barrier dysfunction in an animal model of acute lung injury through the AMPK/SIRT1 pathway [25].

Our finding that irisin is negatively associated with the severity and mortality of sepsis is in line with experimental evidence supporting the anti-inflammatory, antioxidant, and anti-apoptotic effects of irisin. We found that patients who presented higher irisin levels during the first week of sepsis had better outcomes at 28 days after sepsis onset, which supports a beneficial and protective role of irisin against sepsis-induced organ dysfunction, in line with in vitro and in vivo experimental data. We also showed that irisin was negatively associated with most inflammatory biomarkers (CRP, procalcitonin, IL-6, IL-10) at sepsis onset, in accordance with in vitro studies [29,47]. Our finding that relative hypoirisinemia at sepsis onset and during the first week of sepsis is independently associated with sepsis mortality may have important clinical implications, suggesting that exogenous replacement of irisin early in sepsis may be a promising therapeutic intervention.

To date, only experimental data have supported a beneficial role of irisin in sepsis-induced organ injury. Exogenous irisin administration in animal models of sepsis has demonstrated the protective effects of irisin in vivo. Wei et al. showed that irisin treatment suppressed ferroptosis in the liver and lungs of septic mice and markedly reduced serum IL-6 and TNF-a levels. They also showed that irisin treatment attenuated mitochondrial damage and reduced oxidative stress [34]. In another study, the administration of irisin reversed LPS-induced cardiac dysfunction in septic mice [29]. Furthermore, Bi et al. administered irisin intravenously in mice with LPS-induced acute lung injury and showed that irisin remarkably strengthened endothelial barrier function, reducing microvascular leakage [26]. Clinical studies are urgently needed to elucidate the role of irisin in sepsis pathophysiology and explore any therapeutic potential in human sepsis.

Circulating irisin is closely associated with age and BMI, being lower with advancing age and higher with increasing BMI, while weight loss and sarcopenia are associated with lower irisin levels [3,15,50]. We did not find any association of circulating irisin and BMI in our patient cohort. Critically ill patients are bedridden. Lower circulating irisin may be, in part, due to the lack of muscle activity during ICU hospitalization. Also, prolonged critical illness may cause myopathy and polyneuropathy, characterized by muscle weakness and atrophy, which may cause decreased irisin expression [35]. In addition, sepsis is a catabolic state characterized by significant muscle mass loss. It is possible that in the setting of critical illness and sepsis, the profound alterations in muscles are responsible for the lack of association of irisin and BMI, which is normally observed in healthy subjects. Additionally, irisin has been positively associated with the metabolic syndrome, insulin resistance, and HOMA-IR [9]. Nevertheless, in our study, we observed a weak but significant negative association with HOMA-IR upon enrollment. A possible explanation may be that the aberrant inflammatory response during sepsis is responsible for the metabolic dysregulation that enhances insulin resistance. Finally, we detected significant negative correlations of irisin with aminotransferases in accordance with studies depicting a protective effect of irisin against sepsis-induced liver injury [34,47].

In addition to irisin, the adipose tissue and the liver secrete many biologically active proteins (adipokines and hepatokines) that exert immunomodulatory actions (regulatory, pro-inflammatory, and anti-inflammatory) and exhibit alterations during sepsis [51,52]. In particular, adiponectin, leptin, resistin, visfatin, chemerin, and omentin increase at sepsis onset, and their kinetics in the early phase of sepsis are associated with sepsis mortality [53,54,55,56,57]. These findings support the hypothesis that the adipose tissue, the liver, and the muscles are active immunomodulators during sepsis. Furthermore, as new findings help elucidate their role in sepsis, adipokines, hepatokines, and myokines may hold promise for the development of new therapeutic agents for sepsis.

This study is the first to prospectively investigate circulating irisin kinetics in critically ill patients with sepsis and septic shock. The prospective case-control design, the careful selection of age- and gender-matched cases and controls, the appropriate sample, the valid laboratory method for the determination of irisin, and the multivariate analysis with adjustment for confounding factors comprise the main strengths of our study. Yet, we acknowledge certain limitations. An important drawback of the study is the lack of data on physical activity levels of participants in the days prior to sampling. Increased physical activity could augment the production of irisin in the control group. However, this limitation cannot hamper the potential prognostic utility of irisin in cases. Moreover, the control group consisted of healthy subjects and not critically ill patients without sepsis. We also excluded patients who were either dead or discharged before one week of ICU hospitalization, that is, the less and the more severely affected patients. However, the observed mortality rate of our patient cohort is in accordance with the mortality rate reported for sepsis and septic shock in the current consensus definitions based on large patients’ cohorts [27]. Additionally, since this is a single-center study, our findings may not be representative of other populations with sepsis. Finally, it is possible that other unmeasured factors may have confounded our results.

## 5. Conclusions

Irisin kinetics early in sepsis was investigated for the first time in a prospective case-control study on critically ill patients with sepsis and septic shock. We found that circulating irisin levels decreased at sepsis onset compared to healthy controls and increased significantly one week after. Furthermore, irisin was negatively associated with the severity and mortality of sepsis. Finally, we showed that lower circulating irisin levels during the first week of sepsis is an independent predictor of 28-day mortality. These findings are concordant with experimental evidence suggesting a protective role of irisin in sepsis-induced organ dysfunction. This study may have important clinical implications for a therapeutic perspective of irisin in sepsis. More clinical studies are needed to corroborate our findings and to shed light on irisin’s role in sepsis.

## Figures and Tables

**Table 1:** Demographic, clinical, and laboratory baseline parameters * of patients and controls.

Parameters	Patients (N = 102)	Controls (N = 102)	*p*-Value
**Demographic parameters**			
Age ^a^, years	64.7 ± 15.6	66.4 ± 10.3	0.35
Gender, male, n (%)	57 (55.9)	57 (55.9)	0.56
BMI ^a^, kg/m^2^	29.9 ± 8.5	28.1 ± 5.01	0.06
**Clinical parameters**			
APACHE II ^a^	23 ± 7.2	-	-
SOFA ^a^	10 ± 3.3	-	-
Septic shock, n (%)	42 (41.2)	-	-
Death at 28 days, n (%)	30 (29.4)	-	-
**Hematologic and coagulation parameters**			
Hemoglobin ^a^, g/L	93 ± 20	147.9 ± 16.3	<0.001
White Blood Cells ^a^ ×10^9^/L	14.1 ± 8.4	6.97 ± 1.8	<0.001
Platelets ^a^ ×10^9^/L	216.2 ± 118.8	243.8 ± 46.9	0.03
Prothrombin time ^a^, s	14.3 ± 4.7	11.9 ± 0.8	<0.001
aPTT ^a^, s	38.9 ± 9.4	34.4 ± 7.3	<0.001
Fibrinogen ^a^, μmol/L	14.49 ± 5.26	9.06 ± 1.3	<0.001
D-dimer ^a^, μg/L	7278 ± 8158	-	-
**Organ dysfunction biomarkers**			
Lactate ^b^, mmol/L	2.1 (1–9)	-	
Total Protein ^a^, g/L	50 ± 9	71 ± 4.2	<0.001
Albumin ^a^, g/L	24.6 ± 5.9	46.7 ± 5.6	<0.001
Creatinine ^a^, μmol/L	124 ± 71	74 ± 12	0.08
AST ^b^, U/L	33 (8–896)	17.5 (7–34)	<0.001
ALT ^b^, U/L	31 (9–965)	22.5 (9–42)	0.002
**Metabolic parameters**			
Glucose ^a^, mmol/L	7.97 ± 2.9	5.32 ± 1.16	<0.001
HOMA-IR ^b^	8.9 (3.24–34.5)	2.3 (0.65–23.5)	<0.001
**Inflammatory biomarkers**			
CRP ^b^, nmol/L	1257 (67–4104)	32 (1–104)	<0.001
Procalcitonin ^b^, μg/L	0.9 (0.1–100)	-	-
IL-1β ^b^, ng/L	5.9 (5.9–206)	-	-
IL-6 ^b^, ng/L	27.4 (6–444)	-	-
IL-10 ^b^, ng/L	5 (5–300)	-	-
suPAR ^b^, μg/L	13 (2.1–16.8)	-	-
Irisin ^a^, μg/L	22.3 ± 6.8	28.1 ± 6.7	<0.001

* Values of variables are reported as mean ± SD, and those of highly skewed distributed variables are reported as median (range). **Abbreviations:** ALT, alanine aminotransferase; APACHE II, acute physiology and chronic health evaluation score; aPTT, activated partial thromboplastin time; AST, aspartate aminotransferase; BMI, body mass index; CRP, C-reactive protein; HOMA-IR, Homeostasis Model Assessment of Insulin Resistance; IL, interleukin; SOFA, sequential organ failure assessment score; suPAR, soluble urokinase-type Plasminogen Activator Receptor. ^a^ Mean ± SD, ^b^ Median, range.

**Table 2:** Laboratory parameters * of patients with sepsis (N = 60) and septic shock (N = 42), at baseline and one week after enrollment (N = 102).

	**Baseline**			**One Week After**		
	**Sepsis** **(n = 60)**	**Septic Shock** **(n = 42)**	***p*** **-Value**	**Sepsis** **(n = 60)**	**Septic Shock** **(n = 42)**	***p*-Value**
White Blood Cells ^a^ ×10^9^/L	12.5 ± 5.9	16.3 ± 10.7	0.02	8.5 ± 3.2	16.2 ± 11.1	<0.001
Platelets ^a^ ×10^9^/L	230.4 ± 117.6	195.8 ± 118.8	0.15	252.7 ± 120.3	174.6 ± 97.9	0.001
Albumin ^a^, g/L	26 ± 5.6	22.6 ± 5.7	0.004	25.1 ± 4.8	22.5 ± 4.2	0.005
Lactate ^b^, mmol/L	1.2 (1–5)	2.4 (2.1–9)	<0.001	1 (1–2.7)	1.9 (0.7–19)	<0.001
CRP ^b^, nmol/L	848 (67–2076)	1667 (344–4105)	<0.001	524 (76–2686)	962 (124–2410)	0.01
Procalcitonin ^b^, μg/L	0.7 (0.09–47.7)	4.8 (0.14–100)	0.002	0.5 (0.06–15)	1.4 (0.14–83)	0.001
IL-1β ^b^, ng/L	5.9 (5.9–207)	8.8 (5.9–44.8)	0.18	17 (5.9–499)	8.8 (5.9–45)	0.13
IL-6 ^b^, ng/L	16.5 (6–385)	74.4 (10–444)	0.001	25 (4.6–419)	20.5 (6–487)	0.34
IL-10 ^b^, ng/L	5 (5–300)	6.9 (5–87)	0.001	5 (5–300)	5 (5–66)	0.02
suPAR ^b^, μg/L	10.5 (2.2–16.8)	14.1 (4.4–16.8)	0.04	11.3 (2.6–16.8)	12.9 (5.2–16.8)	0.68
Irisin ^a^, μg/L	24.2 ± 7.3	19.6 ± 5.1	<0.001	28.7 ± 10.5	23.6 ± 7	0.004

* Values of normally distributed variables are reported as mean ± SD, and those of non-normally distributed variables are reported as median (range). ^a^ Mean ± SD. ^b^ Median, range. **Abbreviations:** CRP, C-reactive protein; IL, interleukin; suPAR, soluble urokinase-type plasminogen activator receptor.

**Table 3:** Receiver Operator Characteristic Curve Analysis of circulating irisin and inflammatory biomarkers at enrollment to discriminate sepsis from septic shock in 102 patients.

Biomarkers	AUC (95% CI)	*p* Value	Sensitivity	Specificity	Youden Index	Cutoff Value	Positive Predictive Value	Negative Predictive Value
Irisin	0.72 (0.62–0.82)	<0.001	62%	80%	0.42	20.32 μg/L	68.4%	75%
CRP	0.78 (0.68–0.87)	<0.001	80%	69%	0.49	1257 nmol/L	64.4%	83.1%
PCT	0.71 (0.60–0.81)	0.001	60%	83%	0.43	4.30 μg/L	70.9%	74.7%
IL-6	0.69 (0.58–0.79)	0.001	70%	62%	0.32	24.50 ng/L	56.4%	74.7%
IL-10	0.68 (0.57–0.79)	0.003	60%	83%	0.43	5.88 ng/L	70.9%	74.7%
suPAR	0.64 (0.53–0.75)	0.02	75%	59%	0.34	11.79 μg/L	55.9%	77%

**Abbreviations:** AUC, area under the curve; CI, confidence interval; CRP, C-reactive protein; IL, interleukin; PCT, procalcitonin; suPAR, soluble urokinase-type plasminogen activator receptor.

**Table 4:** Receiver Operator Characteristic Curve Analysis to ascertain the optimum cutoff value of irisin and other circulating biomarkers at enrollment to discern death from survival in 102 patients with sepsis.

Biomarkers	AUC (95% C.I.)	*p* Value	Sensitivity	Specificity	Youden’s Index	Cutoff Value	Positive Predictive Value	Negative Predictive Value
Irisin	0.81 (0.69–0.93)	<0.001	77%	93%	0.63	19.92 μg/L	88.5%	86.1%
CRP	0.72 (0.60–0.83)	0.001	67%	74%	0.4	1462 nmol/L	63.9%	75.9%
PCT	0.65 (0.54–0.78)	0.02	53%	76%	0.29	4.60 μg/L	61.3%	70%
IL-6	0.65 (0.53–0.78)	0.02	53%	83%	0.36	110.3 ng/L	68.6%	71.7%
IL-10	0.65 (0.53–0.77)	0.02	64%	84%	0.39	5.68 ng/L	63.7%	74.8%
suPAR	0.55 (0.44–0.67)	0.39 ^‡^	-	-	-	-	-	-

**Abbreviations:** AUC, area under the curve; C.I., confidence interval; CRP, C-reactive protein; IL, interleukin; PCT, procalcitonin; suPAR, soluble urokinase-type plasminogen activator receptor. ^‡^ Non-significant *p*-value.

**Table 5:** Multivariate Cox Regression analysis for the independent predictors of mortality (expressed as quartiles), adjusting for APACHE II score in 102 septic patients.

	b	SE_b_	Wald	df	*p*-Value	HR	95% for C.I.
**Independent predictors at enrollment**							
Irisin	−0.81	0.28	8.41	1	0.004	0.44	0.26–0.77
CRP	−0.008	0.17	0.002	1	0.96	0.99	0.70–1.39
IL-6	0.29	0.18	2.66	1	0.10	1.34	0.94–1.90
APACHE II	0.37	0.19	3.94	1	0.04	1.44	1.01–2.08
**Independent predictors one week after enrollment**							
Irisin	−1.0	0.23	18.24	1	<0.001	0.37	0.23–0.58
CRP	−0.19	0.19	1.11	1	0.29	0.82	0.57–1.19
IL-6	0.52	0.20	6.70	1	0.01	1.68	1.13–2.49
APACHE II	0.77	0.24	9.94	1	0.002	2.16	1.34–3.47

**Abbreviations**: APACHE II, acute physiology, and chronic health evaluation score; b, regression coefficient; C.I., confidence interval; CRP, C-reactive protein; df, degree of freedom; HR, Hazard Ratio; IL-6, interleukin 6; SE_b_, standard error of b.

**Table 6:** Spearman correlation coefficients * of circulating irisin with laboratory biomarkers in 102 patients at enrollment and one week after.

	Enrollment		One Week after	
	r	p	r	p
***Hematologic biomarkers***				
White Blood Cells	**−0.21**	**0.03**	−0.15	0.11
Platelets	0.06	0.53	0.14	0.15
***Coagulation biomarkers***				
Prothrombin time	−0.14	0.14	−0.17	0.09
aPTT	**−0.26**	**0.008**	−0.15	0.12
Fibrinogen	−0.13	0.19	0.000	0.99
D-dimer	0.12	0.4	**0.53**	**0.006**
***Metabolic biomarkers***				
Lactate	**−0.48**	**<0.001**	**−0.29**	**0.003**
AST	**−0.25**	**0.01**	−0.04	0.67
ALT	**−0.23**	**0.01**	0.06	0.49
Albumin	0.11	0.24	0.14	0.15
Creatinine	−0.07	0.48	0.03	0.72
HOMA-IR	**−0.27**	**0.006**	-	-
BMI	−0.07	0.46	-	-
***Inflammatory biomarkers***				
CRP	**−0.21**	**0.03**	0.06	0.54
Procalcitonin	**−0.26**	**0.008**	**−0.21**	**0.03**
IL-1β	−0.09	0.37	0.18	0.06
IL-6	**−0.21**	**0.03**	−0.001	0.99
IL-10	**−0.29**	**0.003**	0.07	0.48
suPAR	−0.005	0.96	0.13	0.19

**Abbreviations:** ALT, alanine aminotransferase; AST, aspartate aminotransferase; aPTT, activated Partial Thromboplastin Time; BMI, Body Mass Index; CRP, C-reactive protein; HOMA-IR, Homeostasis Model Assessment of Insulin Resistance; IL, interleukin; suPAR, soluble urokinase-type plasminogen activator receptor. * Significant correlations are highlighted in bold.

## Data Availability

Data to support the findings of this study are available upon reasonable request.

## Abbreviations

ALT: alanine aminotransferase; AMPK-α, adenosine monophosphate-activated protein kinase-α; APACHE, Acute Physiology and Chronic Health Evaluation; aPTT, activated Partial Thromboplastin Time; ARDS, acute respiratory distress syndrome; AST, aspartate aminotransferase; AUROC, area under the Receiver Operating Characteristic curve; BMI, Body Mass Index; C.I., Confidence Interval; CRP, C-reactive protein; CV, Coefficient of Variation; df, degree of freedom; DRP1, dynamin-related protein 1; ELISA, Enzyme Linked ImmunoSorbent Assay; FNDC5, Fibronectin type III domain-containing protein 5; GPX4, Glutathione peroxidase 4; HOMA-IR, Homeostasis Model Assessment of Insulin Resistance; HR, Hazard Ratio; ICU, Intensive Care Unit; IL, Interleukin; JAK2, Janus kinase 2; JNK, Jun N-terminal kinase; LATS2, Large Tumor Suppressor kinase 2; LPS, lipopolysaccharide; MAPK, mitogen-activated protein kinases; Mst1, macrophage-stimulating 1; NF-kB, nuclear factor kappa B; NLRP3, NOD-like receptor protein 3; Nrf2, nuclear factor-erythroid 2-related factor 2; PPAR-γ, peroxisome proliferator-activated receptor gamma; PCT, procalcitonin; ROC, Receiver Operating Characteristic; SIRT1, sirtuin 1; SD, standard deviation; SEb, standard error of b; SOFA, Sequential Organ Failure Assessment score; suPAR, soluble urokinase-type Plasminogen Activator Receptor; TLR4, Toll-like receptor 4; TNFα, tumor necrosis factor alpha; T2DM, type 2 diabetes mellitus.
